# Syphilitic Brain Disease

**Published:** 1901-12-07

**Authors:** 


### SYPHILITIC BRAIN DISEASE.

In the course of a lecture at the Queen's Square Hospital, delivered on October 22nd, Sir William Gowers drew attention to certain points of much interest in regard to prognosis and treatment in cases of syphilitic disease of the brain. The diagnosis may be right as to the syphilitic nature of the disease, even though as between tumours and arterial disease it may be wrong. In the latter condition the greatest care is necessary in giving a prognosis. There are certain conditions when, for example, a syphilitic arteritis implicates the whole circumference of the vessel, in which restoration is impossible. Whatever may be the precise way in which treatment acts upon syphilitic growths we know that their ultimate fate is to be changed into cicatricical fibrous tissue which will shrink and contract, and thus it is that when an artery is involved in its whole circumference the final result must be an interference with the circulation, depending as to its degree partly on the amount of the disease, partly on the possibility of collateral circulation becoming established. Hence, however confident we may be as to our power of influencing a syphilitic growth, we can by no means be sure of the persistence of the lumen of a syphilitic artery. Moreover, there is always the possibility of the formation of clot in such vessels, and when an artery is so closed its cavity cannot be restored. All this has to be thought of in regard to prognosis, for with our knowledge as to the control which remedies have over syphilitic disease there is no doubt a tendency to allow the nature of the constitutional malady to influence the prognosis favourably without sufficient consideration being given to the particular process to which the symptoms are directly due. When the blood supply is interfered with by an obstruction in an artery the effects, to which the symptoms are due, are of a purely secondary and simple nature, and the fact of syphilis existing cannot influence the prognosis. Coming now to the treatment and consider ing the use of iodide in cases of cerebral syphilis, although this drug must be given, the treatment must be carefully adjusted to the patient's state. One must not forget the power with which iodide is credited of promoting the coagulation of the blood, lest by giving over large doses one might facilitate the formation of a clot in a narrowed vessel. A dose of 15 grains three times a day is large enough to influence such disease if the patient has not been taking it before, and is as much as it is well to give under the circumstances. The lessened susceptibility to a drug, and the lessened therapeutical activity of a drug in those who are accustomed to its use are matters of much interest. To obtain the full influence of iodide for example, and the same is true of mercury, the system must not be accustomed to its presence. It may happen that a moderate dose of iodide, such as 10 grains three times a day, may quickly cause the disappearance of all symptoms of a gumma in the brain, and yet while this same dose is being continued another gumma may develop. Evidently the organisms by which syphilitic disease is produced may become so accustomed to the remedies by which they are usually controlled as to develop in spite of them. Hence the importance of treatment being energetic but also intermittent.

